# Using phenome-wide association studies and the SF-12 quality of life metric to identify profound consequences of adverse childhood experiences on adult mental and physical health in a Northern Nevadan population

**DOI:** 10.3389/fpsyt.2022.984366

**Published:** 2022-10-06

**Authors:** Karen A. Schlauch, Robert W. Read, Stephanie M. Koning, Iva Neveux, Joseph J. Grzymski

**Affiliations:** ^1^Center for Genomic Medicine, Desert Research Institute, Reno, NV, United States; ^2^School of Public Health, University of Nevada, Reno, NV, United States; ^3^Renown Health, Reno, NV, United States

**Keywords:** SF-12 quality of life metric, adverse childhood experiences, phenome-wide association study, public mental health, social determinants of health

## Abstract

In this research, we examine and identify the implications of Adverse Childhood Experiences (ACEs) on a range of health outcomes, with particular focus on a number of mental health disorders. Many previous studies observed that traumatic childhood events are linked to long-term adult diseases using the standard Adverse Childhood Experience Questionnaire. The study cohort was derived from the Healthy Nevada Project, a volunteer-based population health study in which each adult participant is invited to take a retrospective questionnaire that includes the Adverse Childhood Experience Questionnaire, the 12-item Short Form Survey measuring quality of life, and self-reported incidence of nine mental disorders. Using participant’s cross-referenced electronic health records, a phenome-wide association analysis of 1,703 phenotypes and the incidence of ACEs examined links between traumatic events in childhood and adult disease. These analyses showed that many mental disorders were significantly associated with ACEs in a dose-response manner. Similarly, a dose response between ACEs and obesity, chronic pain, migraine, and other physical phenotypes was identified. An examination of the prevalence of self-reported mental disorders and incidence of ACEs showed a positive relationship. Furthermore, participants with less adverse childhood events experienced a higher quality of life, both physically and mentally. The whole-phenotype approach confirms that ACEs are linked with many negative adult physical and mental health outcomes. With the nationwide prevalence of ACEs as high as 67%, these findings suggest a need for new public health resources: ACE-specific interventions and early childhood screenings.

## Introduction

Childhood adversity and trauma have long been associated with greater risk of adult physical and mental health outcomes (1–35). Adverse Childhood Experiences (ACEs) are defined as traumatic events and unsafe environments occurring in children before the age of 18 (3). ACEs include the incidence of emotional, physical, sexual maltreatment, neglect, substance abuse within the household, mental illness in the household, violence, and incarceration of a member in the household (3). These experiences may include unsafe housing, violence, lack of education, or access to food, which are categories of Social Determinants of Health (SDOH) defined by the Center for Disease Control (CDC). SDOH factors have been shown to have strong impact on individuals’ physical health and quality of life: importantly, studies indicate that SDOH alone, as well as when paired with genetics, underlie health and wellbeing (36).

Numerous national studies indicate the seriousness of ACEs with prevalence as high as 67% of adults experiencing at least one ACE and 16% experiencing at least four ACEs. The CDC reports that at least five of the top ten leading causes of death are associated with ACEs (18, 19, 21, 37). Other research highlights the implications and consequences of ACEs, including risk for chronic disease such as asthma, cancer, diabetes, heart disease, inflammatory disorders, obesity and others (4, 6, 9, 10, 18, 19, 21, 38). The relationship between ACEs and mental illness is also known (32, 39–44): relationships with mental disorders such as adult depression, anxiety, eating disorders, schizophrenia, substance abuse, suicide, among others have been clearly established (23–25, 30, 39, 42, 44).

The Healthy Nevada Project (HNP) is an all-comers, adult population health and genomics study based in Northern Nevada. The study also collects self-reported survey information on ACEs and other SDOH. Nevada has a high prevalence of adults with mental illness and poor access to care (e.g., care^1^) and the problem is more acute in children^2^. The state of Nevada has structural healthcare delivery problems and is at or near the bottom in public health funding (45), resulting in an increase in preventable deaths (46). As access to care, and the social and structural determinants of health are all linked to health outcomes, there are unique opportunities in the HNP cohort for studying problems beyond population genomics. Here, a three-pronged approach to study the effects of ACEs is used: we first observe the distribution of ACEs in the cohort with respect to sociodemographic characteristics, as well as self-reported mental health conditions. A more comprehensive examination between ACE exposure and a wide spectrum of mental and physical health outcomes is performed with phenome-wide association analyses (PheWAS): first using ACEs as a predictor in a dose-response style and secondly as a binary case vs. control covariate. Additionally, the SF12-v2^®^ quality of life metric is used to point out quality of life differences between participants with high ACE exposure and those with none. Although we use a different approach compared to previous studies, the HNP cohort follows outcomes published since the original study in 1998 (3).

## Materials and methods

### Data disclosure statement

In order to minimize the chance of individual reidentification for participants in this study, a subset of the phenotype data and summary statistics of phenome-wide association results are available at https://doi.org/10.5061/dryad.73n5tb30g. For data derived from electronic health records (EHR), all HIPAA regulations were followed, including removing individuals with an age greater than 88 years old. For further information, please see our full data availability statement below.

## The Renown electronic health records database

The Renown Health EHR system was instantiated in 2007 on the EPIC system (EPIC System Corporation, Verona, WI, United States), and contains lab results, diagnosis codes (ICD9/ICD10), and demographic information of approximately 1.6 million hospital patient visits from 2005 to the present date.

## IRB and informed consent

This study was conducted under a human subject protocol approved by the University of Nevada Institutional Review Board under project #1106618-15. Participants in the Healthy Nevada Project undergo written and informed consent to having genetic information associated with (EHR) in a deidentified manner. Inclusion criteria are individuals older than 18 years who can complete the consent process either online or in person at a study location. A copy of the consent can be found at https://healthynv.org/about/consent/. Patient identifiers are not incorporated into the research EHR: the EHR and genetic data are linked in a separate environment *via* a unique identifier as approved by the IRB.

## The Healthy Nevada Project cohort

The Healthy Nevada Project (HNP), is a volunteer-based population health study that was formed in 2016 (38, 47–51). As of May 2022, the HNP includes 45,421 whole-exome sequenced participants who are cross-referenced with up to sixteen years of EHR. The HNP also includes a voluntary, retrospective Social Health Determinants Questionnaire containing mental health, social health, and ACE-related questions. More than 18,000 participant responses to the survey have been collected and collated representing approximately 5% of the population of the Reno/Sparks metropolitan area where the study is focused.

This research focuses on the **16,581** HNP participants with self-reported survey responses to the ten adverse childhood events questions. We refer to this as the HNP_ACE_ cohort. Demographics are included in Table 1 and Supplementary Figure 1. Reported ethnicity was based on the genetic admixture from Exome + sequencing of all HNP participants (38).

**TABLE 1:** Distribution of ACEs in the HNP_ACE_.

Num Aces	N	%	African American	East Asian	European	LatinX	South Asian
0	5,677	34.24%	80	183	4,666	483	24
1	3,139	18.93%	58	67	2,538	323	13
2	2,148	12.95%	39	39	1,704	246	3
3	1,637	9.87%	33	34	1,294	185	2
4	1,277	7.70%	29	29	992	164	6
5	921	5.55%	18	15	687	149	2
6	744	4.49%	24	5	564	112	0
7	492	2.97%	11	5	389	59	2
8	336	2.03%	10	4	243	63	0
9	156	0.94%	4	4	117	22	0
10	54	0.33%	6	0	29	15	0
Total	16,581		312	385	13,223	1,821	54

Table represents the distribution of number of ACEs with respect to ethnicity.

## Social Health Determinants Questionnaire

All consenting HNP participants are invited to complete the HNP Social Health Determinants Questionnaire. The survey is voluntary and confidential bound through an NIH Certificate of Confidentiality. It consists of 103 multi-part sociodemographic-related questions including ACEs, other traumatic events, education level, household income, substance abuse, drug use, mental illnesses, and other behavioral patterns.

The HNP ACE questionnaire follows the ten questions from the standard ACE instrument, covering physical, sexual, and emotional abuse, physical and emotional neglect, and household dysfunction, mental illness, incarceration, intimate partner violence, substance abuse, and divorce in the home (3, 18, 19, 52). A standard ACE questionnaire based on the original version can be found here: https://elcentro.sonhs.miami.edu/research/measures-library/aces/aces_eng.doc.docx. The survey was administered to adult participants on the Survey Monkey platform^3^, based on retrospective recall of experiences in the first 18 years of life.

The HNP questionnaire also includes the option of self-reporting any of nine mental illnesses/disorders: Attention Deficit/Hyperactivity Disorder (ADHD), Anxiety, Bipolar Disorder (BP), Depression, Eating Disorder (ED), Obsessive-Compulsive Disorder (OCD), Post-Traumatic Stress Disorder (PTSD), Schizophrenia, and Substance Use Disorder. The wording of the questionnaire for self-reporting the first eight mental conditions is: “Have you ever been diagnosed with, or treated for, any of the conditions.” Substance use disorder is assessed by the question “Substance use disorder: have you ever been addicted to any substance”; for example: nicotine, alcohol, marijuana, cocaine, methamphetamine, LSD/Magic Mushrooms, Ecstasy, prescription stimulants, or painkillers not as prescribed, opium. Possible answers are “Yes” or “No” in both cases.

## ACE cohorts and ACE score

Each of the ten ACEs was self-reported as “Yes” or “No” for each of ten types of events, with unanswered questions left as “NA.” Any participant answering at least one of the ten ACE questions was included in this study; i.e., participants were excluded if all ACE questions were left unanswered. The ACE score was computed as the sum of affirmative responses a participant reported (21, 32, 53, 54). A participant with two ACEs, for example, encountered two different types of ACEs at least one time each by the age of 18. Although the score does not measure event frequency within ACE type, it represents the accumulation of different types of ACEs. For this study, we define the HNP_ACE_ **case cohort** as participants with four or more ACEs, and the **control cohort** as those with no ACEs. This definition is similar to those utilized by Merrick, Jones, and Godoy (18, 19, 55). As reported in (38), question non-response was low: 517 (0.31%) of the questions were unanswered, stemming from 384 participants. The ACE score was computed for these 384 individuals, as all had at least one ACE. Note that ACE subcohorts are defined differently per study. There is no rigid consensus in the literature to define ACE subgroups. For ease of discussion, let ACE_*i*_ denote the group of participants who experience *i* ACEs. Jones and Godoy only examine ACE_4+_ and ACE_0_ (19, 55); Merrick separates participants into ACE_1_, ACE_2–3_, ACE_4+_ (18), and Gilbert divides the study participants into ACE_1–3_, ACE_4–6_, ACE_7–9_ (53). Both Felitti and Dube examine ACE_1_, ACE_2_, ACE_3_ and ACE_4+_ (3, 4). Although the definition of ACE subcohorts is dependent on the sample size, sample demographics, study hypotheses, and other factors, the group “high ACE exposure” is most often constructed as ACE_4+_.

## Phenome-wide association studies

Phenome-wide association studies (PheWAS) examine and identify associations between a large number of disease phenotypes and a specific genotype or phenotype of interest. Here we used the PheWAS approach to identify associations between 1,703 possible disease phenotypes in the HNP_ACE_ and the number of ACEs. Disease phenotypes were based on participants’ recorded EHR ICD codes, which were then aggregated and converted into 1,703 individual phenotype groups (“phecodes”) using the R package “PheWAS” as described in Carroll and Denny (56). Only the phenotype groups that included at least 20 cases were used for downstream analyses, following Carroll’s protocol (56).

**FIGURE 1:**
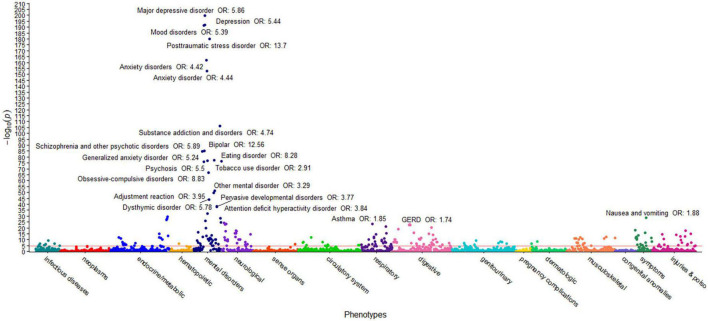
ACEs PheWAS Dose-response ICD10 only. This figure illustrates the results of 1,447 individual associations between the ACE score and the incidence of phenotype group (phecode) of the HNP_ACE_ cohort. Covariates included in the models are sex and age at genotyping. Each point denotes the *p*-value of association of that phenotype. The red horizontal line represents the Bonferroni-corrected significance level of α = 3.5 × 10^–5^. Only associations with *p* < 1 × 10^–20^ are annotated for ease of viewing.

The first PheWAS used the ACE score to assess a dose-response relationship with each of 1,447 disease groups. Specifically, a logistic regression of disease status versus the ACE score was performed for each of the 1,447 phenotypes with more than 20 members, controlling for age and sex. The raw *p*-values for each of the 1,447 associations are shown in Supplementary Table 1; a Bonferroni correction for the number of comparisons yields α = 3.5 × 10^–5^ (0.05/1,447). This value is represented by a horizontal red line in Figure 1. Note that only associations with *p*-value *p* < 1 × 10^–20^ were annotated for ease of viewing.

The second PheWAS used the ACE case/control status as a predictor for disease. Specifically, a logistic regression of disease state versus ACE case/control status was performed for each phenotype group with more than 20 members, controlling for age and sex. There were 1,703 associations conducted. Results are shown in Supplementary Table 2 in which all *p*-values are raw. A Bonferroni correction yields a significance threshold of α = 2.9 × 10^–5^. Again, this value is denoted in Supplementary Figure 2 as a red horizontal line. For ease of viewing, only associations yielding a *p*-value *p* < 1 × 10^–20^ were annotated.

As many of the questionnaire participants had cross-referenced EHR data (*N* = 13,728, 83%), we performed a supplementary set of PheWAS that added the ICD diagnosis codes of the nine mental illnesses for participants who were not diagnosed in the Renown Healthcare system. The details of this analysis and its results are included in the Supplementary material.

## The SF-12 v2^®^ short form survey

The HNP survey recorded responses to the short form SF-12 questions. The SF-12 questions are a shorter one-page version of the SF-36, consisting of 12 of its 36 questions and adaptable to a diverse set of populations (57). Survey responses to SF-12v2 questions were used to generate the mental composite score (MCS) and physical composite score (PCS) as is standard (58). Scores greater than 50 represent a better quality of life compared to the average. The HNP SF-12 survey contained only 1,122 missing answers (0.56%) based on 686 individuals who failed to answer one or more mental health survey questions. Thus, 686 SF-12 agglomerative scores were not computable, and these participants were excluded from further study, leaving 15,895 participants for analyses including both ACE and SF-12 scores (Supplementary Figure 1).

## Statistical analysis

All hypothesis tests were two-sided. All single hypothesis tests were tested at the α = 0.05 significance level. The word “significant” corresponds to a statistically significant result. The “PheWAS” package v. 0.99.5.5 in R was used for both phenome-wide association studies. As SF-12 scores were negatively skewed, either square root function transforms were used with Student *t*-tests, or Mann–Whitney tests were used with means and medians reported, medians reported.

## Results

### Distribution of ACEs in the HNP_ACE_

Results of the 16,581 participants responding to the ACE survey are shown in Table 1: 65.8% of adults recalled at least one type of ACE in childhood, and 24.1% endured four or more different types of ACEs. This distribution is very similar to other studies (3, 12, 19). When compared to other ancestries, Europeans generally were less likely to incur more than four ACEs than African American or LatinX participants. The number of ACE cases was 3,980 and the number of ACE controls was 5,677. Other demographic details of the 16,581 self-reporting participants are presented in Supplementary Table 3, showing that participants with college education have a notably lower prevalence of ACEs than the general cohort. Supplementary Table 4 demonstrates that females incur a higher number of ACEs than males. Supplementary Table 5 shows the percentage of each ACE group in the respective income bracket. Of interest is the lowest income bracket, which shows the starkest increase in participants with greater ACE exposure.

## Distribution of mental health and ACEs in the HNP_ACE_

The distribution of self-reported mental illnesses alongside ACE exposures is shown in Table 2. Note that the HNP_ACE_ results stem strictly from the volunteer questionnaire, whereas the national prevalence is based on clinical diagnoses, specific patient characteristics, and their diagnosis codes.

**TABLE 2:** Self-reported mental illness prevalence in the HNP_ACE_.

Self-reported illness	National prevalence	HNP_ACE_ prevalence	HNP_ACE_ no ACEs	HNP_ACE_ two or more ACEs	HNP_ACE_ four or more ACEs
ADHD	4.4%	5.7%	2.9%	8.4%	10.6%
Anxiety	19.1%	31.4%	18.9%	42.4%	51.0%
Bipolar disorder	2.8%	3.5%	1.0%	5.9%	8.4%
Depression	8.4% ^a^	33.6%	18.5%	46.1%	55.6%
Eating disorder	5%	5.4%	2.6%	8.1%	10.6%
OCD	1.2%	4.0%	1.8%	6.2%	8.5%
PTSD	3.6%	10.4%	3.7%	17.3%	24.8%
Schizophrenia	< 1%	0.43%	0.03%	0.79%	1.1%
Substance Abuse	3.8%	11.0%	5.6%	16.0%	20.0%

This table shows the prevalence of each *self-reported* illness in the HNP_ACE_. ^a^National prevalences are taken from the National Alliance on Mental Illness, https://www.manmi.org/mhstats, 2020; National Institute of Mental Health, https://www.nimh.nih.gov/health/statistics/attention-deficit-hyperactivity-disorder-adhd, 2001–2003, and the National Center for Drug Abuse Statistics, 2022, https://drugabusestatistics.org/. Note that depression is self-reported as “Have you ever been diagnosed with, or treated for Depression”; the national prevalence of depression is referenced here as having at least one major depressive event. Note that we present the prevalence of each mental disorder in three subcohorts: ACE_0_, ACE_2+_ and ACE_4+_. The ACE4 + does not contain ACE_2–3_, and is focused on only high exposure participants. This changes the prevalence notably.

Table 3 outlines that some mental disorders occur at a higher percentage in participants with a higher number of ACEs. For example, in Table 3, 62% of participants with ten ACEs reported as having been diagnosed with PTSD, compared to 3.9% in the ACE controls. In fact, this same trend is shown clearly for bipolar disorder, eating disorder, OCD, PTSD, schizophrenia and substance abuse in this table. Furthermore, Supplementary Table 6 shows an interesting trend: participants who have experienced ACEs show a general increasing trend in the number of concurrent self-reported mental conditions. In other words, if ACE_*i*_ is the group of people reporting *i* ACEs, as *i* increases, the percentage of mental illnesses reported by ACE_*i*_ increases in general. The trend is more noticeable with *i* ≥ 3, as well as in the categories with at least three mental illnesses reported.

**TABLE 3:** ACEs and mental health.

Num Aces	ADHD	Anxiety	BP	Depression	ED	OCD	PTSD	SZ	Substance Abuse
0	2.99%	19.69%	0.99%	19.26%	2.67%	1.83%	3.89%	0.04%	5.83%
1	4.40%	27.55%	1.92%	31.00%	4.07%	2.75%	6.01%	0.27%	8.59%
2	6.17%	34.18%	3.42%	37.56%	5.23%	3.85%	8.85%	0.34%	11.91%
3	7.27%	37.73%	3.99%	40.64%	7.23%	4.91%	12.27%	0.76%	13.68%
4	8.85%	43.98%	4.87%	50.32%	6.76%	6.24%	15.90%	0.33%	16.03%
5	10.00%	47.78%	7.56%	52.33%	10.85%	7.22%	21.98%	1.00%	18.37%
6	11.69%	52.54%	8.85%	55.10%	10.28%	8.68%	25.27%	0.97%	21.05%
7	13.75%	57.17%	10.48%	61.01%	14.88%	10.60%	33.06%	1.68%	24.43%
8	9.20%	64.02%	13.19%	67.17%	11.69%	10.40%	37.20%	2.16%	26.14%
9	13.64%	62.34%	18.83%	72.08%	19.48%	16.88%	46.10%	2.61%	27.27%
10	20.75%	77.36%	22.64%	71.70%	26.42%	22.64%	62.26%	5.66%	26.42%

This table represents the percentage of each ACE group having this mental health disorder. For example, 62.3% of participants reporting 10 ACEs have PTSD. Note that the rows nor columns add up to 100% as many participants had no mental health disorders or ACEs. Note the abbreviations for bipolar disorder (BP), eating disorder (ED), obsessive disorder (OCD), schizophrenia (SZ).

## Phenome-wide association studies

The first PheWAS examined associations between the ACE score and the incidence of 1,447 medical phenotypes. The thirteen most statistically significant associations were with mental disorders, with effect sizes ranging from 0.15 (SE = 0.012) (other mental disorder) to 0.39 (SE = 0.021) (PTSD); thus every additional type of ACE experienced corresponds to an increase in the odds of the diseases 16% and 47%, respectively. Effect sizes of associations with suicide attempt were 0.32 to 0.35, meaning that for each type of ACE endured, the odds of the participant making a suicide attempt increase by 33% on average, with *p*-values ranging from *p* = 8.1 × 10^–32^ to *p* = 3.91 × 10^–18^. These values are reported in Supplementary Table 1, with the strongest associations (*p* < 1 × 10^–20^) shown in Table 4.

**TABLE 4:** Most significant associations of the dose-response phenome-wide association study.

Phenotype Description	Beta	SE	OR	Lower	Upper	*P*-value	N Cases	N Controls
Mood disorders	0.204	0.009	1.226	1.225	1.227	2.00 × 10^–110^	3,592	7,319
Depression	0.201	0.009	1.223	1.222	1.224	1.30 × 10^–104^	3,410	7,319
Major depressive disorder	0.216	0.010	1.241	1.240	1.241	2.93 × 10^–98^	2,391	7,319
Anxiety disorders	0.179	0.009	1.196	1.196	1.197	1.42 × 10^–97^	4,490	7,319
Anxiety disorder	0.181	0.009	1.198	1.198	1.199	1.73 × 10^–92^	3,851	7,319
Tobacco use disorder	0.177	0.009	1.194	1.193	1.195	6.97 × 10^–90^	3,286	9,987
Posttraumatic stress disorder	0.386	0.021	1.472	1.470	1.474	1.30 × 10^–76^	333	7,319
Schizophrenia/ psychotic disorders	0.221	0.013	1.248	1.247	1.249	9.86 × 10^–66^	1,180	7,319
Psychosis	0.218	0.013	1.244	1.243	1.245	7.61 × 10^–63^	1,157	7,319
Bipolar	0.306	0.019	1.358	1.357	1.360	8.05 × 10^–59^	412	7,319
Substance addiction and disorders	0.241	0.015	1.273	1.272	1.274	8.68 × 10^–59^	714	9,987
Generalized anxiety disorder	0.195	0.013	1.216	1.215	1.217	1.70 × 10^–54^	1,261	7,319
Other mental disorder	0.148	0.012	1.159	1.159	1.160	4.61 × 10^–37^	1,692	7,319
Morbid obesity	0.129	0.010	1.138	1.137	1.139	2.15 × 10^–35^	1,908	8,629
Sleep disorders	0.108	0.009	1.115	1.114	1.115	4.10 × 10^–34^	3,253	9,410
Nausea and vomiting	0.105	0.009	1.111	1.110	1.112	4.43 × 10^–34^	2,953	10,772
Alcohol-related disorders	0.202	0.017	1.224	1.223	1.225	5.74 × 10^–34^	608	9,987
Adjustment reaction	0.165	0.014	1.179	1.178	1.180	8.34 × 10^–33^	1,068	7,319
Obesity	0.097	0.008	1.102	1.102	1.103	1.09 × 10^–32^	4,161	8,629
Dysthymic disorder	0.205	0.017	1.227	1.226	1.229	1.49 × 10^–32^	586	7,319
Tooth Diseases	0.161	0.014	1.175	1.174	1.176	1.65 × 10^–32^	958	12,230
Overweight	0.091	0.008	1.095	1.095	1.096	5.23 × 10^–32^	5,096	8,629
Suicidal ideation or attempt	0.322	0.027	1.381	1.378	1.383	8.06 × 10^–32^	175	7,319
Insomnia	0.115	0.010	1.122	1.122	1.123	1.83 × 10^–31^	2,420	9,410
Sleep apnea	0.119	0.010	1.126	1.125	1.127	2.48 × 10^–30^	2,403	9,410
Shortness of breath	0.112	0.010	1.119	1.118	1.119	4.63 × 10^–30^	2,866	6,945
Esophagitis	0.100	0.009	1.105	1.104	1.105	1.24 × 10^–29^	3,498	9,381
Diseases of esophagus	0.099	0.009	1.104	1.103	1.104	2.03 × 10^–29^	3,574	9,381
GERD	0.100	0.009	1.106	1.105	1.106	2.34 × 10^–29^	3,377	9,381
Alcoholism	0.207	0.019	1.230	1.229	1.231	1.41 × 10^–28^	457	9,987
Suicidal ideation	0.348	0.032	1.417	1.414	1.419	6.33 × 10^–28^	127	7,319
Digestive symptoms	0.097	0.009	1.102	1.101	1.102	4.52 × 10^–27^	3,055	9,069
Back pain	0.083	0.008	1.086	1.086	1.087	5.24 × 10^–25^	4,603	9,122
Agoraphobia and Panic Disorder	0.193	0.019	1.213	1.212	1.215	1.78 × 10^–24^	438	7,319
Asthma	0.096	0.009	1.101	1.100	1.101	2.06 × 10^–24^	2,579	9,078
Chronic airway obstruction	0.166	0.016	1.181	1.179	1.182	3.19 × 10^–24^	848	9,078
Migraine	0.100	0.010	1.105	1.105	1.106	2.18 × 10^–23^	1,987	10,586
Chronic pain	0.088	0.009	1.092	1.092	1.093	3.86 × 10^–23^	3,790	7,704
Abdominal pain	0.076	0.008	1.079	1.079	1.080	5.11 × 10^–23^	4,933	8,792
Chronic pain syndrome	0.204	0.021	1.226	1.224	1.227	5.77 × 10^–23^	349	11,946
Neurological disorders	0.126	0.013	1.134	1.133	1.135	1.24 × 10^–22^	1,150	12,224
Electrolyte Disorders	0.094	0.010	1.099	1.098	1.100	1.71 × 10^–22^	2,462	11,180
Obstructive sleep apnea	0.111	0.011	1.117	1.116	1.118	2.25 × 10^–22^	1,917	9,410
Constipation	0.116	0.012	1.122	1.124	1.123	4.83 × 10^–22^	1,282	12,443
Opiates affecting therapy	0.122	0.013	1.128	1.130	1.129	6.64 × 10^–22^	1,218	9,902
Other dyspnea	0.098	0.010	1.102	1.104	1.103	1.52 × 10^–21^	2,709	6,945
Heartburn	0.101	0.011	1.106	1.107	1.106	7.39 × 10^–21^	1,981	9,381

This table shows the strongest statistically significant dose-response associations with ACEs and phenotypes (*p*-values *p* < 1 × 10^–20^).

The second case/control PheWAS examined which EHR phenotype groups associate with high ACE exposure (four or more ACEs) when compared to no ACE exposure. Of the 1,703 phenotype groups containing more than 20 participants, 237 were statistically significant after using the Bonferroni correction (Supplementary Figure 2), with very similar results as the dose-response PheWAS (Supplementary Table 2). Analyses for the phenome-wide analyses which included both ICD codes and self-reported illnesses were performed identically, and results are shown in Supplementary Tables 7–9 and Supplementary Figures 3, 4.

## SF-12 quality of life scores in ACE cases and controls

We observed similar patterns as those in Ware (58), although the means were consistently lower. Physical Functioning and Bodily Pain presented the greatest correlation (0.710). Clear differences were identified in the PCS and MCS between ACE cases and ACE controls. The mean PCS scores were computed as 47.9 for cases and 51.1 for controls; a *t*-test on the transformed values yields *p* < 2.2 × 10^–16^, SE = 0.022. Similarly, the mean MCS of cases and controls was 45.8 and 52.7, respectively, with *p* < 2.2 × 10^–16^, SE = 0.022. Scores for participants suffering from self-reported mental health disorders were also significantly lower than those without a self-reported mental health diagnosis (Supplementary Table 10).

## Discussion

This study observes the distribution of ACEs in an all-comers retrospective survey of almost 17,000 participants. The all-comers population of Healthy Nevada Project comprises an over-representation of female, highly educated, white, and middle-to-high-income individuals, relative to the overall population of the Renown Health service area in Nevada. Despite this, with and without respect to biological sex, ancestry, and income, the distribution of HNP ACEs follow many previous studies, e.g., (3, 18, 19). Here, we focus on the profound effect that childhood trauma has on adult mental health.

We believe our study is novel in its broad examination of all mental diseases with a focus on nine specific disorders as shown in Supplementary Table 9. By analyzing a broad spectrum of mental health outcomes in a single population cohort, we were able to better assess the relative magnitudes of specific mental health burdens likely attributable to high ACEs in our cohort. Unsurprisingly, PheWAS results demonstrate strong associations between ACEs and mental health disorders overall, as expected based on prior research (22, 26, 30, 34, 42, 44, 59). These associations between ACEs and mental health mirror other trauma studies, in which disaster survivors and refugees also demonstrate increased prevalence of anxiety, depression and PTSD due to traumatic past experiences (60–62). Together these results indicate that previous trauma is clearly a major factor in mental health outcomes.

Our study subsequently identified pronounced differences in SF-12 quality of life metrics between the ACE cases and controls, with the two metrics for cases notably under the national mean of 50, while ACE control cohort metrics were above the national mean. Furthermore, we identified significant SF-12 score differences between participants with and without mental disorders (Supplementary Table 10). As annual costs in North America stemming from the incurrence of ACEs was estimated in Bellis et al. to be $748 billion (59), these stark differences in quality of life provide additional evidence that ACEs can be costly both in body and mind.

This study, focused in Northern Nevada, is a window into deeply rooted problems of public health in the United States. A valid concern is whether or not screening for ACEs and studying the long-term health impact of ACEs will bring us any closer to addressing and reducing their health consequences [e.g., (63)] particularly in states such as Nevada with low public health infrastructure. We found a profound association between the number of ACEs endured and increases in suicide attempt. Suicide ideation, attempt, and completion are all on the rise in youth (64). Thus, a practical intervention for individuals with high ACEs may be more focused mental health screening and suicide prevention programs with an important caveat that *post hoc* interventions have been shown to be largely ineffective. For example, Heymann et al. (65) showed no reduction in intimate partner violence (ACEs: abuse in the household) for couples undergoing Couple Commitment and Relationship Enhancement (Couple CARE) for parents of newborns (65). There is a heritability in ACEs either from genetics but more likely from environmental or learned behavior. One area where prevention has shown encouraging results is related to ACEs neglect and specifically food insecurity; numerous studies have shown relieving this has positive impacts on health (e.g., food reducing diabetes risk and healthcare costs) (66). Thus ACE-specific interventions may begin in an area of public health investment given these successes. Recently, the HNP has started working with a Federally Qualified Health Center (FQHC) and a behavioral health and addiction center to focus on preventing ACE occurrences with the overall goal to reduce its prevalence in our community.

Based on the results in this study, ACEs are associated with a number of physical and mental health burdens. However, it is well known that many of these have genetic components (38, 48, 49, 67). Both components must be included to produce a reliable risk assessment for health outcomes. Previous research by Schlauch et al. used an interaction model to examine the effect of ACEs and genetics on BMI and found the phenotypic effect of ACEs be strengthened for those carrying specific germline genetic variations (38). In order to better understand the overall effects of ACEs, particularly with severe mental disorders, this suggests that future studies may want to include phenotypic associations with ACEs, as well as *GxE* interactions to identify missing phenotypic variability that is often unaccounted for in standard analyses.

### Limitations

There are several limitations we can note about our approach. Firstly, only nine mental conditions were self-reported by participants, and as many of these disorders were not diagnosed in the EHR, the second supplementary PheWAS analysis based on ICD codes and the self-reported disorders was biased for these nine conditions. The preliminary PheWAS, however, does not include self-reported mental illnesses, so it is also not quite accurate, although each PheWAS independently shows that ACEs are strongly predictive of mental illness. The sample size of the cohort is somewhat prohibitive when studying rarer disorders and/or high ACE exposure; the HNP is continuously growing, thus this restriction will likely be relieved.

## Data availability statement

The data analyzed in this study are subject to the following licenses/restrictions: These data are available to qualified researchers upon reasonable request and with permission of the Center for Genomic Medicine. EHR data for the Healthy Nevada Project cohort are subject to HIPAA and other privacy and compliance restrictions. Phenotype data for each de-identified participant will be made available on https://doi.org/10.5061/dryad.73n5tb30g, with a small subset of data removed to comply with HIPAA requirements. The Center for Genomic Medicine encourages and collaborates with scientific researchers on an individual basis. Examples of restrictions that will be considered in requests to data access include but are not limited to: (1) Whether the request comes from an academic institution in good standing and will collaborate with our team to protect the privacy of the participants and the security of the data requested. (2) Type and amount of data requested. (3) Feasibility of the research suggested. (4) Amount of resource allocation for the IHI and Renown Hospital required to support the collaboration. Any correspondence and data availability requests should be addressed to Joe.Grzymski@dri.edu and Craig.Kugler@dri.edu.

## Ethics statement

The studies involving human participants were reviewed and approved by University of Nevada Institutional Review Board. The patients/participants provided their written informed consent to participate in this study.

## Author contributions

KS processed and analyzed the data and wrote the manuscript. RR performed data analysis and wrote the manuscript. IN acquired and processed Social Health Determinants Questionnaire data. SK provided critical manuscript review. JG conceived and procured funding for the Healthy Nevada project and this study, had full access to all data in the study, and takes responsibility for the integrity of the data and the accuracy of the data analysis. All authors provided input on the manuscript.

## Footnotes

1
https://www.mhanational.org/issues/ranking-states

2
https://www.mhanational.org/issues/2021/mental-health-america-youth-data

3
www.SurveyMonkey.com
