# Supplementary material for: Using phenome-wide association studies and the SF-12 quality of life metric to identify profound consequences of adverse childhood experiences on adult mental and physical health in a Northern Nevadan population
Source: Front Psychiatry. 2022 Oct 6;13:984366. doi: 10.3389/fpsyt.2022.984366 (PMC9583677; doi:10.3389/fpsyt.2022.984366)
Supplement: Supplementary file 1 [file Data_Sheet_1.DOCX]

**Supplementary Online Content**

**Appendix.** Supplementary Materials and Methods, Results and Discussion, and References

**Figure S1.** ACEs Demographic Flowchart

**Figure S2.** Image of PheWAS results of ACEs Case/Control ICD Only

**Figure S3.** Image of PheWAS results of ACEs Dose-response Self-reported and ICD

**Figure S4.** Image of PheWAS results of ACEs Case/Control Self-reported and ICD

**Table S1.** Summary statistics of the PheWAS ACEs Dose-response ICD Only

**Table S2.** Summary statistics of the PheWAS ACEs Case/Control ICD Only

**Table S3.** ACEs and Education in the HNP_ACE_

**Table S4.** ACEs by Sex in the HNP_ACE_

**Table S5.** ACEs and Income in the HNP_ACE_

**Table S6**. ACEs and Multiple Mental Health Diagnoses in the HNP_ACE_

**Table S7.** Summary statistics of the PheWAS ACEs Dose-response Self-reported and ICD codes

**Table S8.** Summary statistics of the PheWAS ACEs Case/Control Self-reported and ICD codes

**Table S9.** ICD codes of Nine Self-Reported Mental Illnesses and their Prevalence

**Table S10.** Mental Health Diagnosis and SF-12 Score in the HNP_ACE_

This supplementary material has been provided by the authors to give readers additional information about this work.

***Materials and Methods***

***PheWAS for ICD codes and self-reported mental disorders***

Analyses for the phenome-wide association studies which included both ICD codes and self-reported illnesses were performed identically, and results are shown in Supplemental Figures S2-S3. Again, 1,447 and 1,703 association tests were conducted, respectively, with Bonferroni corrections *α* = 3.5 x 10^-5^ and *α* = 2.9 x 10^-5^.  The results tables of these two additional PheWAS are also included in the Supplemental Tables S7-S8.

***ICD Codes of Survey Mental Illnesses/Conditions***

Ideally, a self-reported mental health condition would be recorded in the EHR with the corresponding ICD code (clinical diagnosis), indicating that the patient and clinician (if the patient was seen in the Renown system) were concomitant. In the HNP_ACE_ cohort there were 6,645 unique participants with self-reported mental disorders who did not have a corresponding recorded clinical ICD code. In these cases, the ICD code for the disease was added for the PheWAS only; all other tables or results include only the self-reported survey results. As this study focuses on *self-reported* events and disease, we believe that this intervention may yield a better representation of the HNP_ACE_. The questionnaire mental conditions were generally described: “Anxiety”, “Depression“, “Bipolar disorder - manic-depression“, “Schizophrenia“, “Attention-deficit/hyperactivity disorder (ADHD)“, “Post Traumatic Stress Disorder (PTSD)“, “Obsessive-Compulsive Disorder (OCD)“, and “Eating disorder“. The ICD code system is very detailed, thus attaching an ICD code to these self-reported mental conditions is imprecise. To correct for this, the ICD code most prevalent in participants with cross-referenced ICD codes for that specific illness was used. For example, all survey participants who self-reported “Schizophrenia” were diagnosed most commonly with the EHR ICD10 code F20. Thus, each participant self-reporting Schizophrenia without a recorded ICD value (59 in this case) was assigned the ICD code F20 to perform the PheWAS. The weakness of this approach is that only nine illnesses/conditions were self-reported by participants, thus the addition of the ICD codes for just these nine conditions biased the entire distribution of phenotype incidences. Additionally, using the identical general ICD code for each participant is likely imprecise, but at the very least provides an estimate of diagnoses.

***Results and Discussion***

***PheWAS of ACE Case vs. ACE Control using ICD codes only***

This phenome-wide association study was performed using a series of logistic regressions as described in the Methods section. The case/control association to morbid obesity was notable: the odds ratio of morbid obesity was OR=2.24, 95% CI= [2.21, 2.23], *p*-value=1.78 x 10^-31^. As in the previous PheWAS, a number of inflammatory-related illnesses were linked with high ACE exposure: asthma, other respiratory illnesses, diabetes, liver diseases, among others. The twelve most statistically significant associations were with mental disorders, with odds ratios in the interval [2.11, 27.74]. The greatest odds ratios of significant associations include Borderline Personality Disorder (OR=27.74, 95% CI=[26.50, 29.06], *p*-value=5.92x10^-6^), suicidal ideation (OR=9.40, 95% CI=[9.26, 9.55], *p*-value=1.13x10^-19^), and PTSD (OR=10.75, 95% CI=[10.63, 10.87], *p*-value=7.23 x 10^-40^).

***PheWAS of ACE Case vs ACE Control with ICD codes and self-reported mental disorders***

Upon the addition of ICD codes of those who self-reported one or more of the nine mental conditions, but were not clinically diagnosed at Renown Health, the odds ratio of PTSD was OR=13.70, 95% CI= [13.62, 13.78], *p*-value=1.21x10^-180^, and that of schizophrenia was OR=30.25, 95% CI=[29.37, 31.16], *p*-value=5.87x10^-13^. Bipolar disorder was also more pronounced in the ACE cases, with OR=12.56, CI=[12.46,12.66], and *p*-value=4.13x10^-86^.

***PheWAS of ACE Score with ICD codes and self-reported mental disorders***

Unsurprisingly, there is an increase of the dose-response for the nine mental disorders for which the ICD codes were artificially added. Highlighted results show much lower *p*-values, and greater effect sizes for all nine disorders.

All PheWAS results speak for themselves: the results tables and figures clearly indicate that by far the greatest disorders affected by having more ACEs than less are mental disorders: suicidal ideation and/or attempt, schizophrenia, alcoholism, PTSD, mood disorders, psychosis, and many others. Additionally, obesity, chronic pain, asthma, GERD, and other physical disorders seem to be affected by the number of ACEs incurred in childhood.

***SF-12 Quality of Life scores***

The SF-12 is a universally implemented questionnaire that measures self-reported health-related quality of life (HRQOL). Its origin is the SF-36, a 36-question Short-Form Health Survey generated by the Medical Outcomes Study ^1^, that consists of 36 questions which can be used to assess health-related quality of life (HRQOL) irrespective of cohort characteristics. The SF-36 and SF-12 have been validated as reliable HRQOL metrics in many health studies, including both mental ^2-4^ and physical disorders ^5-8^.

The shorter SF-12v2® consists of 12 questions that span the same targeted health components as the SF-36; however, due to its much shorter length, it is well-suited for populations with shortened attention spans, and/or mental illness ^2^. The SF-12v2’s 12 questions are aggregated into the same eight components that the SF-36 examines: Physical Functioning, Role Physical, Bodily Pain, General Health, Vitality, Social Functioning, Role Emotional, Mental Health. The MCS is a sum of all eight components, weighted heavier for Vitality, Social Functioning, Role Emotional, and Mental Health; whereas the PCS is weighted heavier for Physical Functioning, Role Physical, Bodily Pain, General Health, and Vitality. Mental composite score (MCS) and physical composite score (PCS) for each participant were calculated similarly to Ware ^9^. Canonically, the mean PCS and mean MCS scores are used to compare general quality of life metrics across case/control cohorts ^9-12^. These non-age and sex adjusted scores are based on a US sample of 7,069 subjects collected and studied in 1998. Additional details are presented in Ware, 1998 Table 8.2 and Ware, 2002, Tables 11.1 - 11.23, as well as in other references ^1,13-15^.

Our results also indicate that participants suffering from a mental health disorder showed decreased MCS and PCS scores when compared to the control cohort (Supplemental Table S10).

***A special note on schizophrenia***

We also note that 42/3980 (0.11%) ACE cases have schizophrenia, whereas only 0.035% (2/5677) controls self-report the disease. This significant disparity results in an odds ratio of 30 [7.89, 253.72], with *p*= 2.6 x 10^-14^ upon a simple Fisher’s Exact test. Clearly, this is underpowered, as the general prevalence of schizophrenia is rare, but does produce an interesting trend.

**References**

1. Ware J, Kosinski M, Keller SD. A 12-Item Short-Form Health Survey: construction of scales and preliminary tests of reliability and validity. *Med Care*. 1996;34(3):220-233. doi:10.1097/00005650-199603000-00003.

2. Huo T, Guo Y, Shenkman E, Muller K. Assessing the reliability of the short form 12 (SF-12) health survey in adults with mental health conditions: a report from the wellness incentive and navigation (WIN) study. *Health Qual Life Outcomes*. 2018;16(1):34-38. doi:10.1186/s12955-018-0858-2.

3. Stansfeld SA, Roberts R, Foot SP. Assessing the Validity of the SF-36 General Health Survey. *Quality of Life Research*. 1997;6(3):217-224.

4. Vilagut G, Forero CG, Pinto-Meza A, et al. The mental component of the short-form 12 health survey (SF-12) as a measure of depressive disorders in the general population: results with three alternative scoring methods. *Value Health*. 2013;16(4):564-573. doi:10.1016/j.jval.2013.01.006.

5. Druss BG, Rohrbaugh RM, Levinson CM, Rosenheck RA. Integrated medical care for patients with serious psychiatric illness: a randomized trial. *Arch Gen Psychiatry*. 2001;58(9):861-868. doi:10.1001/archpsyc.58.9.861.

6. Hagell P, Westergren A. Measurement properties of the SF-12 health survey in Parkinson's disease. *J Parkinsons Dis*. 2011;1(2):185-196. doi:10.3233/JPD-2011-11026.

7. Jenkinson C, Layte R, Jenkinson D, et al. A shorter form health survey: can the SF-12 replicate results from the SF-36 in longitudinal studies? *J Public Health Med*. 1997;19(2):179-186. doi:10.1093/oxfordjournals.pubmed.a024606.

8. Ruotolo I, Berardi A, Sellitto G, et al. Criterion Validity and Reliability of SF-12 Health Survey Version 2 (SF-12v2) in a Student Population during COVID-19 Pandemic: A Cross-Sectional Study. *Depress Res Treat*. 2021;2021:6624378. doi:10.1155/2021/6624378.

9. Ware J, Kosinski M, Keller S. *SF-12: How to Score the SF-12 Physical and Mental Health Summary Scales*. Boston, MA: The Health Assessment Lab, New England Medical Center; 1998.

10. Jafari A, Nejatian M, Momeniyan V, Barsalani FR, Tehrani H. Mental health literacy and quality of life in Iran: a cross-sectional study. *BMC Psychiatry*. 2021;21(1):499–11. doi:10.1186/s12888-021-03507-5.

11. Larson CO, Schlundt D, Patel K, Beard K, Hargreaves M. Validity of the SF-12 for use in a low-income African American community-based research initiative (REACH 2010). *Prev Chronic Dis*. 2008;5(2):A44.

12. Singh A, Gnanalingham K, Casey A, Crockard A. Quality of life assessment using the Short Form-12 (SF-12) questionnaire in patients with cervical spondylotic myelopathy: comparison with SF-36. *Spine (Phila Pa 1976)*. 2006;31(6):639-643. doi:10.1097/01.brs.0000202744.48633.44.

13. Fleishman JA, Selim AJ, Kazis LE. Deriving SF-12v2 physical and mental health summary scores: a comparison of different scoring algorithms. *Qual Life Res*. 2010;19(2):231-241. doi:10.1007/s11136-009-9582-z.

14. Farivar SS, Cunningham WE, Hays RD. Correlated physical and mental health summary scores for the SF-36 and SF-12 Health Survey, V.I. *Health Qual Life Outcomes*. 2007;5(1):54-58. doi:10.1186/1477-7525-5-54.

15. Ware JE, Kosinski M, Keller S. *SF-36 Physical and Mental Health Summary Scales: a User's Manual*. Boston, MA: The Health Assessment Lab, New England Medical Center; 1994.

**Figure S1. ACEs Demographic Flowchart**

**
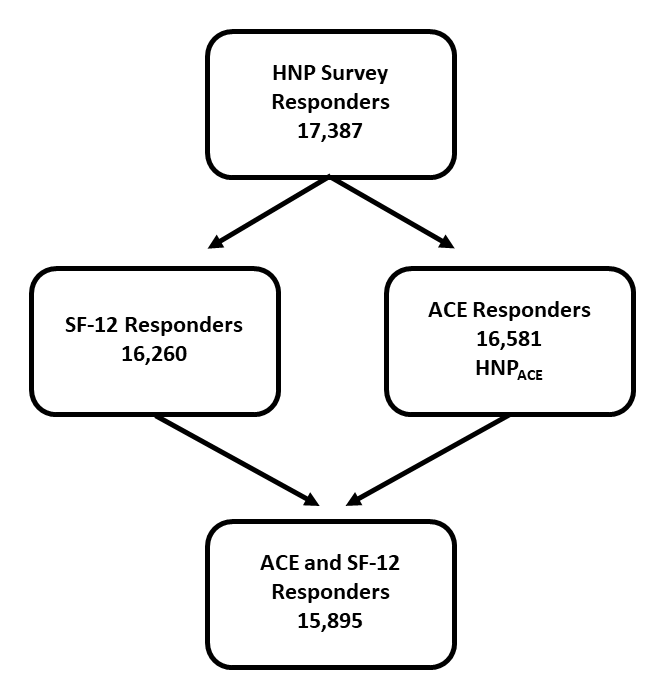
**

Figure S1. The HNP cohort who answered the social health questionnaire (*N*=17,387) was first parsed for participants who answered at least one of ten ACE questions (*N*=16,581). This sub-cohort was called the HNP_ACE_.  The original cohort was then parsed for participants who answered all SF-12 questions (*N*=16,260). The sub-cohort that contained participants with ACE exposures and SF-12 scores was formed last, with *N*=15,895.

**Figure S2. ACEs Case/Control ICD Only**


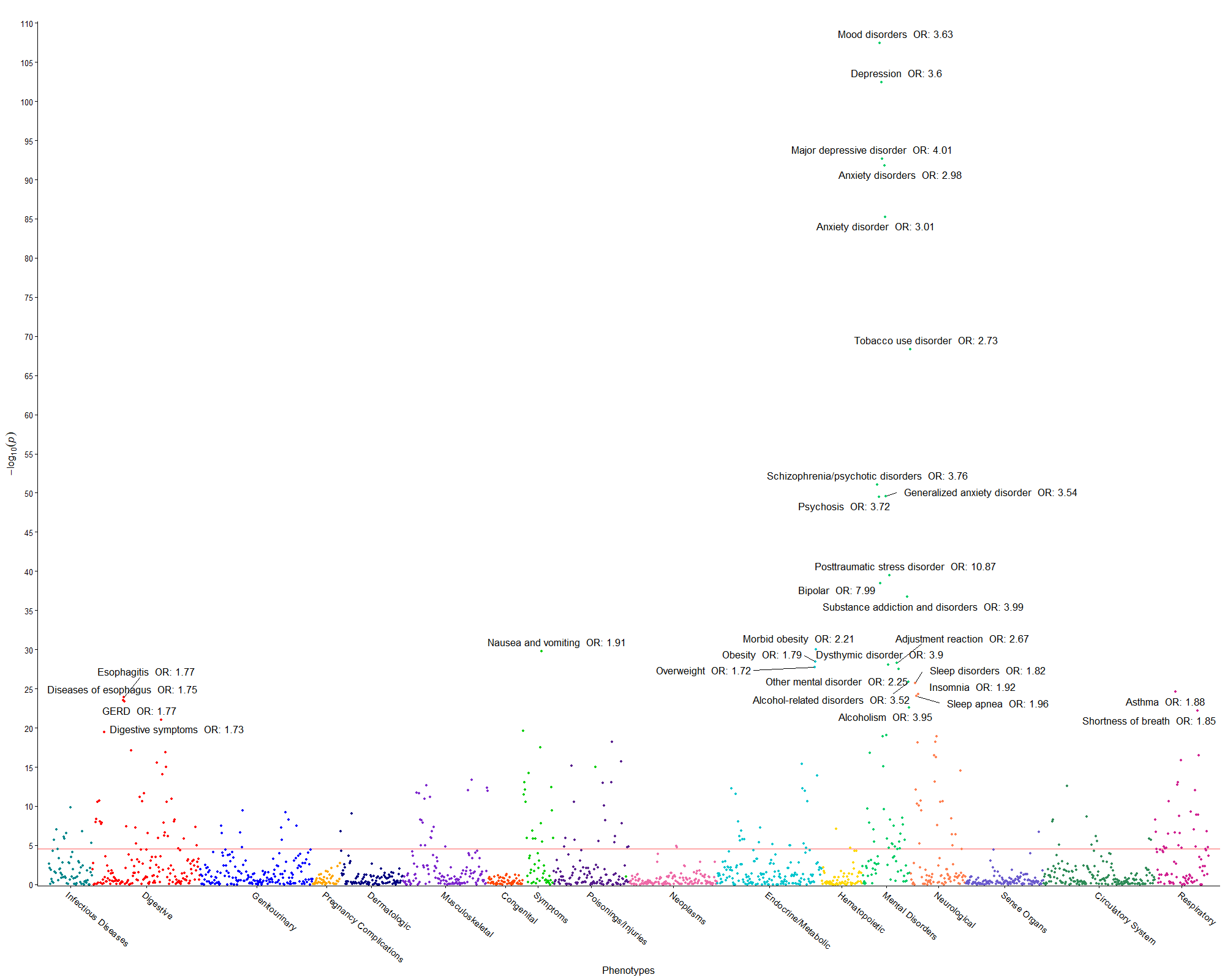


Figure S2. This figure presents the results of 1,703 associations of the case vs. control PheWAS to predict the incidence of phenotype group. Covariates include age at genotyping and sex. Each point denotes the *p*-value of association of that phecode. The red horizontal line represents the Bonferroni-corrected significance level of *α* = 3.5 x 10^-5^. Only associations with *p* < 1x10^-20^ are annotated for ease of viewing.

**Figure S3. ACEs Dose-response Self-reported and ICD**


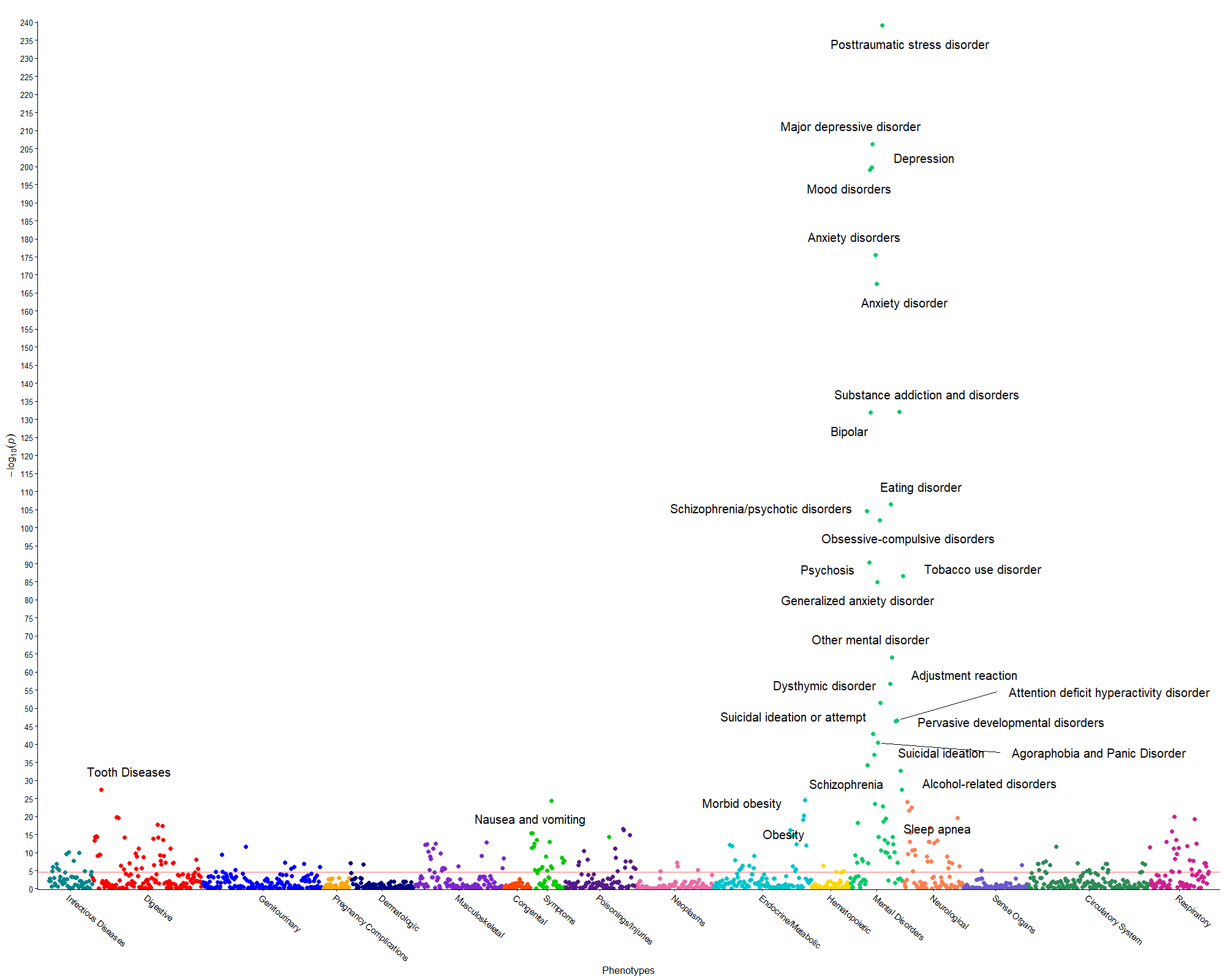


Figure S3. This figure illustrates the results of 1,447 individual associations between the number of ACEs a participant endured and phenotype groups (phecodes) of the HNP_ACE_ cohort. This PheWAS includes both the ICD codes of all survey participants, and the incidences of the self-reported illnesses Anxiety, ADHD, Bipolar Disorder, Depression, Eating Disorder, OCD, PTSD, Schizophrenia, and Substance Abuse. Covariates included in the models are sex and age at genotyping. Each point denotes the *p*-value of association of that phecode. The red horizontal line represents the Bonferroni-corrected significance level of *α* = 3.5 x 10^-5^. Only associations with *p* < 1x10^-20^ are annotated for ease of viewing.

**Figure S4. ACEs Case/Control Self-reported and ICD**


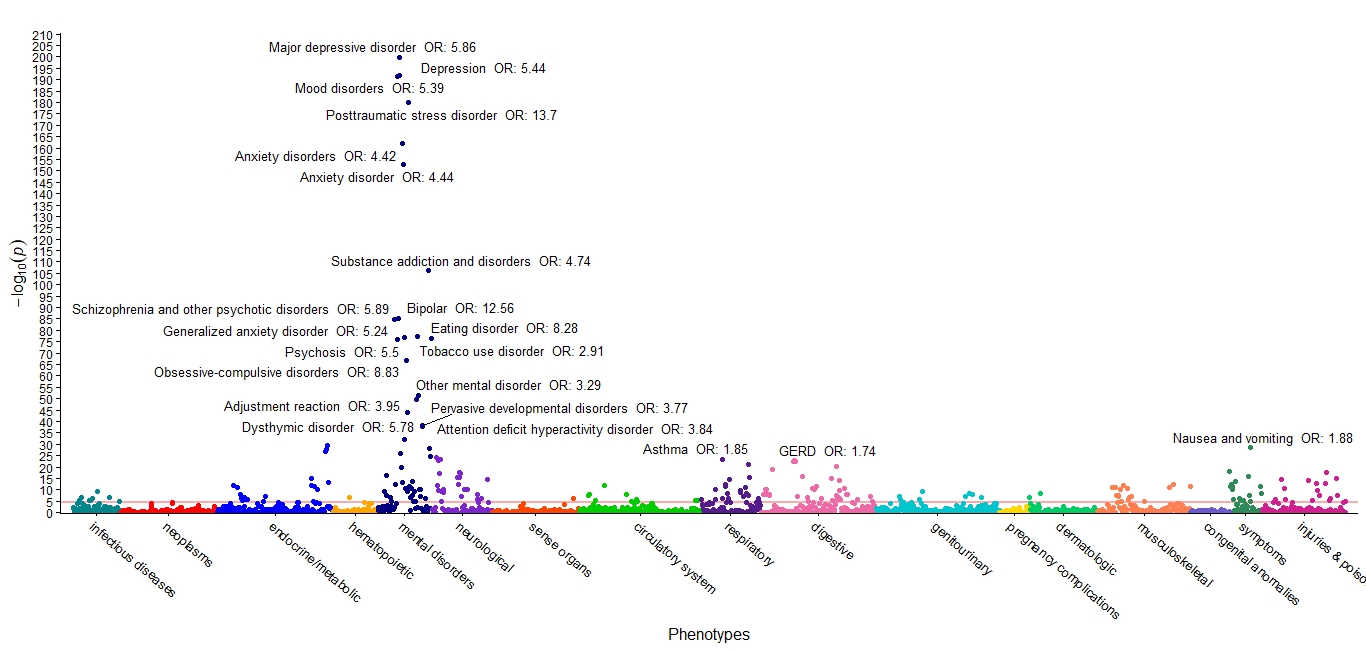


Figure S4. This figure presents the results of 1,703 associations of the case vs. control PheWAS to predict the incidence of phenotype group. Covariates include age at genotyping and sex. This PheWAS includes both the ICD codes of all survey participants, and the incidences of the self-reported illnesses Anxiety, ADHD, Bipolar Disorder, Depression, Eating Disorder, OCD, PTSD, Schizophrenia, and Substance Abuse. Each point denotes the *p*-value of association of that phecode. The Bonferroni significance level is *α* = 2.9 x 10^-5^, represented by the red horizontal line. Only associations with *p* < 1x10^-20^ are annotated for ease of viewing.

**Table S1. PheWAS ACEs Dose-response ICD Only**

Table S1. This table shows the significant results from 1,447 associations using the ACE score to predict the incidence of each phenotype group (phecode). Covariates in the logistic model are sex and age at genotyping. The Bonferroni significance level is *α* = 3.5 x 10^-5^. All *p*-values in the table are raw. Also included is the effect size (β, Beta) of the regression, the standard error (SE), the odds ratio (OR), and 95% confidence intervals. Each phecode group contains at least 20 cases (N cases). The full results table for this pheWAS can be found at https://datadryad.org/stash/share/7V7r5wkigb5qtHJXo6UVgWNtdoMCA90r4Dn79gGSgJE

**Table S2. PheWAS ACEs Case/Control ICD Only**

Table S2. This table shows the significant results from 1,703 associations using the ACE score to predict the incidence of each phenotype group (phecode). Covariates in the logistic model are sex and age at genotyping. The Bonferroni significance level is *α* = 2.9 x 10^-5^. All *p*-values in the table are raw. Also included is the effect size (β, Beta) of the regression, the standard error (SE), the odds ratio (OR), and 95% confidence intervals. Each phecode group contains at least 20 cases (N cases). The full results table for this pheWAS can be found at https://datadryad.org/stash/share/7V7r5wkigb5qtHJXo6UVgWNtdoMCA90r4Dn79gGSgJE

**Table S3. ACEs and Education in the HNP_ACE_**

| **College Degree, Including Associate’s Degrees** | | |
| --- | --- | --- |
| **num Aces** | **N** | **% of HNP_ACE_** |
| 0 | 3,896 | 23.50% |
| 1 | 2,067 | 2.47% |
| 2 | 1,318 | 7.95% |
| 3 | 976 | 5.89% |
| 4 | 730 | 4.40% |
| 5 | 484 | 2.91% |
| 6 | 355 | 2.14% |
| 7 | 237 | 1.43% |
| 8 | 152 | 0.92% |
| 9 | 73 | 0.44% |
| 10 | 18 | 0.11% |
| **Total** | **10,306** |  |

Table S3. This table presents the number and percentage of HNP_ACE_ participants with a college education in each ACE group.

**Table S4. ACEs by Sex in the HNP_ACE_**

|  | **% of ACE_i_ who are Female** | **% of ACE_i_ who are Male** |
| --- | --- | --- |
| **num Aces** | **Female** | **Male** |
| 0 | 64% | 36% |
| 1 | 69% | 31% |
| 2 | 71% | 29% |
| 3 | 73% | 27% |
| 4 | 76% | 24% |
| 5 | 76% | 24% |
| 6 | 78% | 22% |
| 7 | 84% | 16% |
| 8 | 84% | 16% |
| 9 | 84% | 16% |
| 10 | 89% | 11% |

Table S4. This table shows the distribution of females and males in the HNP_ACE_ for each ACE group.

**Table S5. ACEs and Income in the HNP_ACE_**

| **num Aces** | **<$35k** | **$35k-$50k** | **$50k-$75k** | **$75-$100k** | **>$100k** | **Total** | **Percent of ACE_i_ who are in this income bracket** | | | | |
| --- | --- | --- | --- | --- | --- | --- | --- | --- | --- | --- | --- |
|  |  |  |  |  |  |  | **<$35k** | **$35k-$50k** | **$50k-$75k** | **$75-$100k** | **>$100K** |
| 0 | 593 | 734 | 1,024 | 1,117 | 2,060 | 5,528 | 10.73% | 13.28% | 18.52% | 20.21% | 37.26% |
| 1 | 372 | 429 | 624 | 621 | 1,032 | 3,078 | 12.09% | 13.94% | 20.27% | 20.18% | 33.53% |
| 2 | 262 | 305 | 437 | 447 | 655 | 2,106 | 12.44% | 14.48% | 20.75% | 21.22% | 31.10% |
| 3 | 260 | 263 | 338 | 279 | 462 | 1,602 | 16.23% | 16.42% | 21.10% | 17.42% | 28.84% |
| 4 | 220 | 191 | 300 | 218 | 324 | 1,253 | 17.56% | 15.24% | 23.94% | 17.40% | 25.86% |
| 5 | 170 | 179 | 185 | 168 | 203 | 905 | 18.78% | 19.78% | 20.44% | 18.56% | 22.43% |
| 6 | 132 | 150 | 155 | 135 | 161 | 733 | 18.01% | 20.46% | 21.15% | 18.42% | 21.96% |
| 7 | 110 | 85 | 101 | 89 | 101 | 486 | 22.63% | 17.49% | 20.78% | 18.31% | 20.78% |
| 8 | 80 | 63 | 78 | 52 | 56 | 329 | 24.32% | 19.15% | 23.71% | 15.81% | 17.02% |
| 9 | 41 | 16 | 34 | 25 | 39 | 152 | 26.97% | 10.39% | 22.08% | 16.23% | 25.32% |
| 10 | 21 | 9 | 6 | 7 | 11 | 54 | 38.89% | 16.67% | 11.11% | 12.96% | 20.37% |

Table S5. This table lists the first the number of participants in each ACE group belonging to the respective income group. It also shows the income percentage distribution of each ACE group.

**Table S6. ACEs and Multiple Mental Health Diagnoses**

|  | **Mental Health Disorders** |  |  |  |  |  |  |  |  |  |
| --- | --- | --- | --- | --- | --- | --- | --- | --- | --- | --- |
| **Num of ACEs** | 0 | 1 | 2 | 3 | 4 | 5 | 6 | 7 | 8 | 9 |
| 0 | 68.01% | 16.54% | 10.09% | 3.84% | 1.09% | 0.28% | 0.09% | 0.04% | 0.02% | 0.00% |
| 1 | 54.89% | 21.63% | 14.08% | 5.83% | 2.42% | 0.73% | 0.38% | 0.00% | 0.03% | 0.00% |
| 2 | 47.02% | 20.95% | 17.64% | 8.61% | 3.91% | 1.21% | 0.37% | 0.28% | 0.00% | 0.00% |
| 3 | 41.05% | 22.24% | 19.30% | 9.53% | 5.19% | 1.89% | 0.61% | 0.12% | 0.06% | 0.00% |
| 4 | 35.47% | 20.99% | 20.83% | 12.22% | 5.79% | 3.05% | 1.10% | 0.47% | 0.08% | 0.00% |
| 5 | 31.16% | 20.74% | 19.33% | 12.70% | 8.69% | 4.34% | 2.50% | 0.22% | 0.33% | 0.00% |
| 6 | 29.30% | 17.74% | 16.94% | 18.28% | 9.14% | 5.24% | 3.09% | 0.13% | 0.13% | 0.00% |
| 7 | 23.17% | 15.65% | 19.51% | 18.50% | 11.99% | 6.30% | 3.05% | 1.42% | 0.41% | 0.00% |
| 8 | 21.43% | 11.90% | 18.75% | 20.83% | 16.67% | 6.85% | 2.38% | 1.19% | 0.00% | 0.00% |
| 9 | 16.03% | 13.46% | 17.95% | 19.23% | 14.10% | 11.54% | 2.56% | 2.56% | 1.92% | 0.64% |
| 10 | 9.26% | 14.81% | 12.96% | 22.22% | 12.96% | 12.96% | 5.56% | 3.70% | 3.70% | 1.85% |

Table S6. This table is unique in that it combines the number of mental conditions a participant reported with the number of ACEs reported. Each entry denotes the percentage of that ACE group with the respective number of mental conditions.

**Table S7. PheWAS ACEs Dose-response Self-reported and ICD**

Table S7. This table shows the significant results from 1,447 associations using the ACE score to predict the incidence of each phenotype group (phecode). Covariates in the logistic model are sex and age at genotyping. This PheWAS includes both the ICD codes of all survey participants, and the incidences of the self-reported illnesses Anxiety, ADHD, Bipolar Disorder, Depression, Eating Disorder, OCD, PTSD, Schizophrenia, and Substance Abuse. The Bonferroni significance level is *α* = 3.5 x 10^-5^. All *p*-values in the table are raw. Also included is the effect size (β, Beta) of the regression, the standard error (SE), the odds ratio (OR), and 95% confidence intervals. Each phecode group contains at least 20 cases (N cases). The full results table for this pheWAS can be found at https://datadryad.org/stash/share/7V7r5wkigb5qtHJXo6UVgWNtdoMCA90r4Dn79gGSgJE

**Table S8. PheWAS ACEs Case/Control Self-reported and ICD**

Table S8. This table shows the significant results from 1,703 associations using the status of case or control to predict the incidence of phenotype group. Covariates include age at genotyping and sex. This PheWAS includes both the ICD codes of all survey participants, and the incidences of the self-reported illnesses Anxiety, ADHD, Bipolar Disorder, Depression, Eating Disorder, OCD, PTSD, Schizophrenia, and Substance Abuse. The Bonferroni significance level is *α* = 2.9 x 10^-5^. All resulting *p*-values are raw. Also included is the effect size (β) of the regression, the standard error (SE), the odds ratio (OR), and 95% confidence intervals. Each phecode group contains at least 20 cases. The full results table for this pheWAS can be found at https://datadryad.org/stash/share/7V7r5wkigb5qtHJXo6UVgWNtdoMCA90r4Dn79gGSgJE

**Table S9. ICD codes of Nine Self-Reported Mental Illnesses and their Prevalence**

**A. Table of self-reported mental disorders self-reported in the of HNP_ACE_.**

| **Phenotype** | **N self-reported without an ICD diagnosis** | **Total self-reported in survey** | **Percentage not diagnosed in EHR** | **ICD code most frequent** |
| --- | --- | --- | --- | --- |
|  |  |  |  |  |
| ADHD | 736 | 951 | 77.5% | 314.01 |
|  |  |  |  |  |
| Anxiety | 2,643 | 5,199 | 50.8% | F41.9 |
|  |  |  |  |  |
| Bipolar Disease | 360 | 572 | 62.9% | F31.9 |
|  |  |  |  |  |
| Depression | 3588 | 5565 | 64.5% | F32.9 |
|  |  |  |  |  |
| Eating Disorder | 843 | 896 | 94.1% | 307.50 |
|  |  |  |  |  |
| OCD | 619 | 669 | 92.6% | 300.3* |
|  |  |  |  |  |
| PTSD | 1472 | 1735 | 84.8% | F43.10 |
|  |  |  |  |  |
| Schizophrenia | 59 | 71 | 83.1% | F20 |
|  |  |  |  |  |
| Substance Addiction | 1551 | 1818 | 85.3% | F11.20 |

Supplementary Table 9A. This Table shows the number of HNP_ACE_ participants who self-reported a mental disorder, and the number of them who were diagnosed clinically in the Renown EHR system.

**B. Relevant ICD codes of HNP_ACE_ participants with clinically diagnosed mental disorders.**

| **ADHD** | **Anxiety** | **Bipolar Disease** | **Depression** | **Eating Disorders** | **OCD** | **PTSD** | **Schizophrenia** | **Substance Abuse** |
| --- | --- | --- | --- | --- | --- | --- | --- | --- |
| 314.00 | 293.84 | 296.00 | 296.20 | 307.1 | 300.3* | 309.81 | 295.10 | 292.0 |
| 314.01* | 300 | 296.02 | 296.21 | 307.50* | F42 | F43.10* | 295.20 | 292.12 |
| 314.9 | 300.0 | 296.03 | 296.22 | 307.51 | F42.2 | F43.11 | 295.30 | 292.2 |
| F90.1 | 300.00 | 296.05 | 296.23 | 307.52 | F42.8 | F43.12 | 295.34 | 292.8 |
| F90.9 | 300.09 | 296.10 | 296.24 | 307.54 | F42.9 |  | 295.40 | 292.84 |
|  | 300.10 | 296.40 | 296.25 | 307.59 |  |  | 295.7 | 292.85 |
|  | 300.5 | 296.41 | 296.26 | F50 |  |  | 295.70 | 292.89 |
|  | 300.89 | 296.42 | 296.30 | F50.00 |  |  | 295.90 | 292.9 |
|  | 300.9 | 296.44 | 296.31 | F50.01 |  |  | F20* | 304.00 |
|  | 313.1 | 296.45 | 296.32 | F50.02 |  |  | F20.0 | 304.01 |
|  | 313.21 | 296.46 | 296.33 | F50.2 |  |  | F20.1 | 304.02 |
|  | 313.3 | 296.50 | 296.34 | F50.8 |  |  | F20.9 | 304.03 |
|  | F41.0 | 296.51 | 296.35 | F50.81 |  |  | F21 | 304.10 |
|  | F41.3 | 296.52 | 296.36 | F50.82 |  |  | F25.0 | 304.11 |
|  | F41.8 | 296.53 | F32.0 | F50.89 |  |  | F25.1 | 304.12 |
|  | F41.9* | 296.54 | F32.1 | F50.9 |  |  | F25.9 | 304.13 |
|  | F48.8 | 296.55 | F32.2 |  |  |  | F60.5 | 304.30 |
|  | F48.9 | 296.56 | F32.3 |  |  |  | V11.0 | 304.31 |
|  | F99 | 296.60 | F32.8 |  |  |  |  | 304.33 |
|  | R45.2 | 296.61 | F32.9* |  |  |  |  | 304.40 |
|  | R45.5 | 296.62 | F33.0 |  |  |  |  | 304.41 |
|  |  | 296.63 | F33.1 |  |  |  |  | 304.43 |
|  |  | 296.64 | F33.2 |  |  |  |  | 304.60 |
|  |  | 296.65 | F33.3 |  |  |  |  | 304.63 |
|  |  | 296.66 | F33.8 |  |  |  |  | 304.80 |
|  |  | 296.7 | F33.9 |  |  |  |  | 304.83 |
|  |  | 296.80 |  |  |  |  |  | 304.90 |
|  |  | 296.82 |  |  |  |  |  | 304.91 |
|  |  | 296.89 |  |  |  |  |  | 304.92 |
|  |  | F30.10 |  |  |  |  |  | 304.93 |
|  |  | F30.12 |  |  |  |  |  | 305.20 |
|  |  | F30.13 |  |  |  |  |  | 305.21 |
|  |  | F30.3 |  |  |  |  |  | 305.22 |
|  |  | F30.8 |  |  |  |  |  | 305.23 |
|  |  | F30.9 |  |  |  |  |  | 305.40 |
|  |  | F31.0 |  |  |  |  |  | 305.41 |
|  |  | F31.1 |  |  |  |  |  | 305.43 |
|  |  | F31.10 |  |  |  |  |  | 305.50 |
|  |  | F31.11 |  |  |  |  |  | 305.51 |
|  |  | F31.12 |  |  |  |  |  | 305.52 |
|  |  | F31.13 |  |  |  |  |  | 305.53 |
|  |  | F31.2 |  |  |  |  |  | 305.60 |
|  |  | F31.30 |  |  |  |  |  | 305.63 |
|  |  | F31.31 |  |  |  |  |  | 305.70 |
|  |  | F31.32 |  |  |  |  |  | 305.71 |
|  |  | F31.4 |  |  |  |  |  | 305.72 |
|  |  | F31.5 |  |  |  |  |  | 305.73 |
|  |  | F31.60 |  |  |  |  |  | 305.90 |
|  |  | F31.61 |  |  |  |  |  | 305.92 |
|  |  | F31.62 |  |  |  |  |  | 305.93 |
|  |  | F31.63 |  |  |  |  |  | 648.3 |
|  |  | F31.64 |  |  |  |  |  | 648.31 |
|  |  | F31.70 |  |  |  |  |  | 648.33 |
|  |  | F31.71 |  |  |  |  |  | 965.00 |
|  |  | F31.72 |  |  |  |  |  | 965.01 |
|  |  | F31.73 |  |  |  |  |  | F11.10 |
|  |  | F31.74 |  |  |  |  |  | F11.20* |
|  |  | F31.75 |  |  |  |  |  | F11.21 |
|  |  | F31.76 |  |  |  |  |  | F11.229 |
|  |  | F31.77 |  |  |  |  |  | F11.23 |
|  |  | F31.78 |  |  |  |  |  | F11.282 |
|  |  | F31.81 |  |  |  |  |  | F11.29 |
|  |  | F31.89 |  |  |  |  |  | F11.90 |
|  |  | F31.9* |  |  |  |  |  | F11.988 |
|  |  | F32.81 |  |  |  |  |  | F11.99 |
|  |  |  |  |  |  |  |  | F12.10 |
|  |  |  |  |  |  |  |  | F12.188 |
|  |  |  |  |  |  |  |  | F12.20 |
|  |  |  |  |  |  |  |  | F12.21 |
|  |  |  |  |  |  |  |  | F12.90 |
|  |  |  |  |  |  |  |  | F12.920 |
|  |  |  |  |  |  |  |  | F12.922 |
|  |  |  |  |  |  |  |  | F12.929 |
|  |  |  |  |  |  |  |  | F12.951 |
|  |  |  |  |  |  |  |  | F12.988 |
|  |  |  |  |  |  |  |  | F12.99 |
|  |  |  |  |  |  |  |  | F13.10 |
|  |  |  |  |  |  |  |  | F13.20 |
|  |  |  |  |  |  |  |  | F13.21 |
|  |  |  |  |  |  |  |  | F13.230 |
|  |  |  |  |  |  |  |  | F13.239 |
|  |  |  |  |  |  |  |  | F13.282 |
|  |  |  |  |  |  |  |  | F13.90 |
|  |  |  |  |  |  |  |  | F13.94 |
|  |  |  |  |  |  |  |  | F14.10 |
|  |  |  |  |  |  |  |  | F14.129 |
|  |  |  |  |  |  |  |  | F14.90 |
|  |  |  |  |  |  |  |  | F14.929 |
|  |  |  |  |  |  |  |  | F15.10 |
|  |  |  |  |  |  |  |  | F15.129 |
|  |  |  |  |  |  |  |  | F15.188 |
|  |  |  |  |  |  |  |  | F15.20 |
|  |  |  |  |  |  |  |  | F15.21 |
|  |  |  |  |  |  |  |  | F15.23 |
|  |  |  |  |  |  |  |  | F15.90 |
|  |  |  |  |  |  |  |  | F15.920 |
|  |  |  |  |  |  |  |  | F15.929 |
|  |  |  |  |  |  |  |  | F15.93 |
|  |  |  |  |  |  |  |  | F15.988 |
|  |  |  |  |  |  |  |  | F16.188 |
|  |  |  |  |  |  |  |  | F17.203 |
|  |  |  |  |  |  |  |  | F17.209 |
|  |  |  |  |  |  |  |  | F17.213 |
|  |  |  |  |  |  |  |  | F17.218 |
|  |  |  |  |  |  |  |  | F17.219 |
|  |  |  |  |  |  |  |  | F17.228 |
|  |  |  |  |  |  |  |  | F17.229 |
|  |  |  |  |  |  |  |  | F17.293 |
|  |  |  |  |  |  |  |  | F17.298 |
|  |  |  |  |  |  |  |  | F17.299 |
|  |  |  |  |  |  |  |  | F18.10 |
|  |  |  |  |  |  |  |  | F18.90 |
|  |  |  |  |  |  |  |  | F19.10 |
|  |  |  |  |  |  |  |  | F19.20 |
|  |  |  |  |  |  |  |  | F19.21 |
|  |  |  |  |  |  |  |  | F19.230 |
|  |  |  |  |  |  |  |  | F19.239 |
|  |  |  |  |  |  |  |  | F19.90 |
|  |  |  |  |  |  |  |  | F19.929 |
|  |  |  |  |  |  |  |  | F19.930 |
|  |  |  |  |  |  |  |  | F19.939 |
|  |  |  |  |  |  |  |  | F19.94 |
|  |  |  |  |  |  |  |  | F19.951 |
|  |  |  |  |  |  |  |  | F19.959 |
|  |  |  |  |  |  |  |  | F19.982 |
|  |  |  |  |  |  |  |  | F19.988 |
|  |  |  |  |  |  |  |  | F19.99 |
|  |  |  |  |  |  |  |  | F55.2 |
|  |  |  |  |  |  |  |  | O99.320 |
|  |  |  |  |  |  |  |  | O99.321 |
|  |  |  |  |  |  |  |  | O99.322 |
|  |  |  |  |  |  |  |  | O99.323 |
|  |  |  |  |  |  |  |  | O99.324 |

Supplementary Table 9B. This table shows all ICD9 and ICD10 codes of the HNP_ACE_ participants who were clinically diagnosed as one or more of the nine mental disorders of focus. The ICD code in each column with an asterisk denotes the most prevalent ICD code, and was assigned to each participant who self-reported the illness but was not diagnosed at the Renown Health system. These ICD codes were used to complete the second series of phenome-wide association studies.

**Table S10. Mental Health Diagnosis and SF-12 Score**

|  | **N** | **Mean SF-12 MCS** | **Median SF-12 MCS** | **Mean SF-12 PCS** | **Median SF-12 PCS** | **MCS Mann Whitney Test P-value** | **PCS Mann Whitney Test P-value** | |
| --- | --- | --- | --- | --- | --- | --- | --- | --- |
| ADHD | 921 | 44.98 | 46.32 | 48.42 | 51.51 | p < 2.2 x 10^-16^ | P = 3.3 x 10^-13^ | |
| Anxiety | 5029 | 44.94 | 46 | 48.37 | 51.17 | p < 2.2 x 10^-16^ | p < 2.2 x 10^-16^ | |
| BP | 557 | 39.79 | 39.66 | 45.04 | 47.13 | p < 2.2 x 10^-16^ | p < 2.2 x 10^-16^ | |
| Depression | 5388 | 44.83 | 45.85 | 48.13 | 50.67 | p < 2.2 x 10^-16^ | p < 2.2 x 10^-16^ | |
| Eating Disorder | 865 | 43.64 | 44.18 | 47.33 | 50.07 | p < 2.2 x 10^-16^ | p < 2.2 x 10^-16^ | |
| OCD | 643 | 42.56 | 43.16 | 46.86 | 50.27 | p < 2.2 x 10^-16^ | p < 2.2 x 10^-16^ | |
| PTSD | 1689 | 43.42 | 43.98 | 45.85 | 48.22 | p < 2.2 x 10^-16^ | p < 2.2 x 10^-16^ | |
| Schizophrenia | 67 | 41.45 | 40.92 | 43.16 | 43.29 | p = 3.327 x 10^-15^ | p = 2.8 x 10^-13^ | |
| Substance Abuse | 1749 | 46.53 | 48.08 | 46.88 | 49.62 | p < 2.2 x 10^-16^ | p < 2.2 x 10^-16^ | |
| Controls | 8038 | 53.06 | 54.7 | 51.4 | 54.36 |  |  |  |

Table S10. This table reports the mean and median SF-12 physical and mental component scores for each of the groups of participants reported one of the nine mental illnesses. It also includes the Mann-Whitney hypothesis *p*-value.
